# Supplementary material for: The spatial separation of processing and transport functions to the interior and periphery of the Golgi stack
Source: eLife. 2018 Nov 30;7:e41301. doi: 10.7554/eLife.41301 (PMC6294550; doi:10.7554/eLife.41301)
Supplement: Supplementary file 1. — The official full name of ManII is from MGI while the rest are from HGNC. Except GalT and ManII, all names are official symbols. [file elife-41301-supp1.docx]

**Supplementary file 1**

Mouse Genome Informatics (MGI) and Human Genome Organization Gene Nomenclature Committee (HGNC) official full names of glycosylation enzymes used in this study. The official full name of ManII is from MGI while the rest are from HGNC. Except GalT and ManII, all names are official symbols.

| name | GenBank Accession No.: | official full name |
| --- | --- | --- |
| β3GalT6 | BC082998 | beta-1,3-galactosyltransferase 6 |
| β4GalT3 | BC009985.2 | beta-1,4-galactosyltransferase 3 |
| β4GalT7 | NM_007255 | beta-1,4-galactosyltransferase 7 |
| GALNT1 |  | polypeptide N-acetylgalactosaminyltransferase 1 |
| GALNT2 |  | polypeptide N-acetylgalactosaminyltransferase 2 |
| GalT | HG765101 | beta-1,4-galactosyltransferase 1 |
| MGAT1 | M61829 | mannosyl(alpha-1,3-)-glycoprotein beta-1,2-N-acetylglucosaminyltransferase |
| MGAT2 | BC006390 | mannosyl(alpha-1,6-)-glycoprotein beta-1,2-N-acetylglucosaminyltransferase |
| MGAT4B | AB000624 | alpha-1,3-mannosyl-glycoprotein 4-beta-N-acetylglucosaminyltransferase B |
| Man1B1 | BC006079.1 | mannosidase alpha class 1B member 1 |
| ManII | BC138372 | mannosidase2, alpha 1 |
| POMGNT1 | NM_017739 | protein O-linked mannose N-acetylglucosaminyltransferase 1 (beta 1,2-) |
| ST6Gal1 | BC040009 | ST6 beta-galactoside alpha-2,6-sialyltransferase 1 |
| SLC35C1 | NM_018389 | solute carrier family 35 member C1 |
| TPST1 | NM_003596.3 | tyrosylprotein sulfotransferase 1 |
| TPST2 | NM_001008566.1 | tyrosylprotein sulfotransferase 2 |
