# Supplementary material for: The spatial separation of processing and transport functions to the interior and periphery of the Golgi stack
Source: eLife. 2018 Nov 30;7:e41301. doi: 10.7554/eLife.41301 (PMC6294550; doi:10.7554/eLife.41301)
Supplement: Supplementary file 2. [file elife-41301-supp2.docx]

**Supplementary file 2**

Review of previous EM literature that directly addressed lateral localizations of Golgi residents and secretory cargos in comparison with this study.

| Golgi proteins | | this study | literature | | |
| --- | --- | --- | --- | --- | --- |
|  |  | localization | localization | results or data | reference |
| machinery components | COPI (β-COP) | rim and surrounding | rim and surrounding | interior (15), coated buds (116) and vesicles (89) (labeling density, gold particles/µm^2^) | Table 2 (Orci et al., 1997) |
|  | Giantin | rim | interior | interior (58 %), rim + coated buds + vesicles (42 %) (percentage of gold particles) | Table II (Martinez-Menarguez et al., 2001) |
|  |  |  | rim | interior (< 5%), rim + coated buds + vesicles (45 %) (percentage of gold particles) | Fig. 1A,B (Koreishi et al., 2013) |
|  | KDEL receptor | rim and surrounding | rim and surrounding | interior (36 %), rim + coated buds + vesicles (64 %) (percentage of gold particles) | Table II (Martinez-Menarguez et al., 2001) |
|  |  |  | rim | rim to interior ratio = 2.83 (number of gold particles) | Table III (Cosson et al., 2002) |
|  |  |  | rim and surrounding | interior (81), coated buds (160) and vesicles (103) (labeling density, gold particles/µm^2^) | Table 2 (Orci et al., 1997) |
|  | GS27 (membrin) | rim and surrounding | rim | rim to interior ratio = 4.6 (number of gold particles) | Table I (Cosson et al., 2005) |
|  | GS15 | rim | rim | rim to interior ratio = 2.1 (number of gold particles) | Table I (Cosson et al., 2005) |
| Golgi enzymes | Man1B1 | rim | rim | rim to interior ratio > 1.5 (linear density of gold particles) | Fig. 2b,c,e (Rizzo et al., 2013) |
|  | ManII | interior | interior | interior (91), coated buds (17) and vesicles (7) (labeling density, gold particles/µm^2^) | Table I (Orci et al., 2000) |
|  |  |  | interior | interior (69 %), rim + coated buds and vesicles (31 %) (percentage of gold particle) | Table II (Martinez-Menarguez et al., 2001) |
|  |  |  | interior | rim to interior ratio = 0.79 (number of gold particles) | Table III (Cosson et al., 2002) |
|  |  |  | rim | localization to cisternal perforated zone | Fig. 4 (Kweon et al., 2004) |
|  |  |  | interior | rim to interior ratio = 0.79 (number of gold particles) | Table I (Cosson et al., 2005) |
|  | MGAT1 (NAGTI) | interior | interior | interior (214), coated buds (24) or vesicles (17) (in label density, gold particles/µm^2^) | Table I (Orci et al., 2000) |
|  | ST6Gal1 | interior | rim | cisternal perforated zone | Fig. 4 (Kweon et al., 2004) |
|  | GalT | interior | rim | The ratio of the cisternal perforated zone to interior is 2.16:1 (linear density of gold particles). | Table 2 and Fig. 4 (Kweon et al., 2004) |
|  |  |  | interior | rim to interior ratio = 0.55 (number of gold particles) | Table I (Cosson et al., 2005) |
| Secretory cargo | VSVG | interior | interior | interior (1.34), coated buds (0.2) and vesicles (0.16) (linear density of gold particles) | Table I(Mironov et al., 2001) |
|  |  |  | interior | interior (94 %), rim + coated buds + vesicles (6 %) (percentage of gold particle total labeling) | Table II (Martinez-Menarguez et al., 2001) |
|  | FM4 soluble aggregate | rim | rim | rim connecting magavesicles | Fig.4 and 5 (Volchuk et al., 2000) |
