# Supplementary material for: The spatial separation of processing and transport functions to the interior and periphery of the Golgi stack
Source: eLife. 2018 Nov 30;7:e41301. doi: 10.7554/eLife.41301 (PMC6294550; doi:10.7554/eLife.41301)
Supplement: Supplementary file 4. [file elife-41301-supp4.docx]

**Supplementary file 4**

Protocol for en face averaging and radial mean intensity profile

I install the following three macros in Fiji (Plugins->Macros->Install).

1. Macro “Gyradius and intensity normalization.ijm”
2. Macro “Golgi mini-stack alignment.ijm”
3. Macro “Radial mean intensity profile.ijm”

II Steps to average en face view images:

1. Acquire 2D multi-color images of Golgi mini-stacks. Giantin must be co-stained.
2. Crop an en face view of a Golgi mini-stack in a square in Fiji.
3. Subtract the background so that all background pixel values are 0.
4. Save it into a working folder.
5. Select Giantin channel image.
6. Launch the macro “gyradius and intensity normalization.ijm” in Fiji.
7. The macro prompts you to choose the directory to export processed images; once it is selected, the macro runs. A Log window appears to display processing information. The multi-channel image is split into individual channel images and their sizes and intensities are normalized. These processed images are saved in the directory specified by the macro.
8. The procedure can be repeated to process more images of Golgi mini-stacks.
9. Open all images of a specific Golgi marker that are processed by the macro “gyradius and intensity normalization.ijm”.
10. Convert them to an image stack in Fiji.
11. Launch the macro “Golgi mini-stack alignment.ijm” and Golgi mini-stacks are aligned to the center of the canvas.
12. The en face averaged Golgi mini-stack image is acquired by using z-projection in Fiji (Image->Stacks->Z projection).

III Steps to generate radial mean intensity profile:

1. Open the en face averaged image of a Golgi marker.
2. Launch the macro “Radial mean intensity profile.ijm”.
3. In the Results window, copy the first (distance from the center; in pixel) and fourth (mean intensity) column to a spreadsheet processing software, such as Excel and Origin.
4. Determine the radius of the half maximum of the outer slope.
5. The normalized radius is calculated as the radius of a Golgi marker divided by that of corresponding Giantin.
